# Annual Trends of High Tibial Osteotomy: Analysis of an Official Registry in Italy

**DOI:** 10.3390/medicina60071168

**Published:** 2024-07-19

**Authors:** Umile Giuseppe Longo, Alessandro Mazzola, Stefano Campi, Giuseppe Salvatore, Vincenzo Candela, Carlo Casciaro, Diana Giannarelli, Margaux D’Hooghe, Rocco Papalia

**Affiliations:** 1 Fondazione Policlinico Universitario Campus Bio-Medico, Via Alvaro del Portillo, 200, 00128 Rome, Italy; alessandro.mazzola@unicampus.it (A.M.); s.campi@policlinicocampus.it (S.C.); g.salvatore@policlinicocampus.it (G.S.); v.candela@policlinicocampus.it (V.C.); c.casciaro@unicampus.it (C.C.); r.papalia@policlinicocampus.it (R.P.); 2 Research Unit of Orthopaedic and Trauma Surgery, Department of Medicine and Surgery, Università Campus Bio-Medico di Roma, Via Alvaro del Portillo, 21, 00128 Rome, Italy; 3 Facility of Epidemiology and Biostatistics, Fondazione Policlinico Univeristario A. Gemelli, IRCCS, 00168 Rome, Italy; diana.giannarelli@gmail.com; 4 Department of Medicine, University of Navarra, 31008 Pamplona, Spain; margauxdhooghe@gmail.com

**Keywords:** osteoarthritis, knee, tibial, osteotomy, sports medicine, epidemiology, trends

## Abstract

*Background and Objectives:* Knee osteoarthritis is a serious burden for modern countries. Timing of surgery and treatment choice are still a matter of controversy in the orthopedic literature. The purpose of this study was to ascertain the incidence and hospitalization trends of high tibial osteotomy in Italy from 2001 to 2016. *Materials and Methods:* Data are sourced from the National Hospital Discharge Reports (SDO) of the Italian Ministry of Health between 2001 and 2016. *Results:* A total of 34,402 high tibial osteotomies were performed over the study period in Italy. The cumulative incidence was 3.6 cases per 100,000 residents. The age classes 50–54, 55–59 showed the higher number of procedures. In pediatric patients (0–19 years), high tibial osteotomies are also largely performed. The majority of patients having surgery were men with a M/F ratio of 1.5. The mean age of patients was 44.2 ± 19.2 years. Males were significantly younger than females (43.3 ± 20.7 vs. 45.6 ± 17.7). The average length of hospitalization was 6.1 ± 7.3 days. Over the course of the analysis, a declining trend in hospital stay length was seen. The main primary diagnosis codes were “Varus knee” (736.42 ICD-9-CM code, 33.9%), “Osteoarthrosis, localized, primary, leg region” (715.16 ICD-9-CM code, 9.5%). *Conclusions:* Over the study period, high tibial osteotomies in Italy almost halved. Varus deformity and knee osteoarthritis are the leading causes requiring high tibial osteotomy. Except for the pediatric setting, results showed that from the 20–24 age class to the 50–54 age class, there was an increasing request for knee osteotomy, whereas in those aged >60 years, the incidence progressively decreased. The evident decline in HTO performed over the years in Italy seems to reflect a minor role for knee osteotomy in the management of knee OA, as it seems to be primarily reserved for younger male patients.

## 1. Introduction

Knee osteoarthritis (OA) is becoming extremely common in modern countries. Symptomatic OA of the knee affects 14 million patients in the USA, more than half of whom are under the age of 65 [1]. Tricompartmental knee OA is generally managed with total knee arthroplasty (TKA). However, tricompartmental arthritis is significantly less common than single compartmental disease (17% vs. 50%, respectively) [2].

Patients with unicompartmental knee OA who need surgical intervention have increased over time due to changes in demography and physical activity attitudes [3,4]. Treatment options for unicompartmental OA or osteonecrosis include tibial or femoral osteotomy and unicompartmental knee arthroplasty (UKA) [5,6,7,8,9]. In clinical practice, the patient’s age, level of physical activity, and severity of deformity play a role in the treatment choice [3,8,10,11,12]. The two procedures address pain relief by using different biomechanical principles. While keeping the other compartments’ native knee kinematics, UKA replaces the weight-bearing surface of a single osteoarthritic compartment [13]. Conversely, knee osteotomy, by changing joint surface alignment, underloads the chondropathic compartment and corrects the angular knee deformity that led to arthritis [14].

In general, osteotomies can be monoplanar or biplanar, opening-wedge or closing-wedge. Knee osteotomies can be divided into high tibial osteotomy (HTO) and distal femoral osteotomy (DFO). The most popular procedures worldwide are medial opening-wedge HTO for the treatment of varus knee deformity and lateral closing-wedge DFO for the treatment of valgus knee deformity [15].

Possible complications following HTO can be found intraoperatively or postoperatively and are divided into osteo-ligament, infectious, vascular–nervous, hardware-related, and malalignments [16,17,18,19]. Osteo-ligament complications include fractures, intra-articular or related to the osteotomy site (hinge fractures). The latter were classified by Takeuchi et al. into three types [20]. In type I, the fracture line is an extension of the osteotomy line and is proximal or at the height of the proximal tibiofibular joint. In type II, the fracture reaches the distal portion of the proximal tibiofibular joint. Type III corresponds to fractures of the lateral tibial plateau. Delayed union and pseudarthrosis of the osteotomy site are possible, especially in opening osteotomies. High or low patella and changes in the tibial slope (excess or defect) are other possible osteo-ligament complications. Infections can be divided into superficial, deep, related to the hardware or the bone graft. Vasculo-nervous complications include thromboembolic disease (deep vein thrombosis and pulmonary embolism), paralysis of the common peroneal nerve or its branches (in osteotomies involving the fibula or proximal tibiofibular joint), lesions of the popliteal or anterior tibial artery. Hardware-related complications include intolerance (discomfort) to the implanted device, loosening, breakage of the device or screws. Finally, malalignments, which can be assessed by calculating the mechanical axis on an x-ray of the lower limb under load, can be divided into loss of correction, overcorrection, and undercorrection.

When comparing knee osteotomy to UKA, the first showed improved postoperative ROM, higher rates of return to sports, bone stock preservation, and better functional outcomes [21]. In contrast, the latter is burdened by fewer complication rates than knee osteotomy, such as peroneal palsy, deep vein thrombosis, reduced postoperative pain, and lower revision rates [22].

Over time, the increasing popularity of TKA and UKA has led to a decline in the need for knee osteotomies in developed countries [23,24]. However, because of better functional results, symptomatic, physically demanding, young, and active patients with radiographical evidence of mild to moderate knee OA are still recognized as the ideal candidates for knee osteotomy [25,26,27].

Despite the abundance of literature regarding outcomes of knee osteotomies and knee arthroplasty, there are few studies dealing with the incidence trends of HTO over the years. This registry-based study’s aim was to evaluate the incidence of HTO carried out in Italy between 2001 and 2016.

## 2. Materials and Methods

### 2.1. Study Design and Selection of Study Participants

The National Hospital Discharge Records (SDO), an official database made available by the Italian Ministry of Health and encompassing data from all Italian private and public hospitals, served as the source for the analysis of the current study. The International Classification of Diseases, Ninth Revision, Clinical Modification (ICD-9-CM) is used to code diagnoses. The following main procedure code was used to define HTO: 77.87 ICD-9-CM code. Data about individual research subjects are accessible from 2001 to 2016. The patient’s gender, age, place of residence, hospitalization area, length of stay, primary diagnoses, and primary procedures are all included in these anonymized data. In order to determine the prevalence of HTO in Italy, population data from the National Institute for Statistics (ISTAT) were used for each year. Additionally, the incidence rates were stratified by year, age class, and gender. All patients included in the present study underwent an HTO even though the main procedure code was related to other knee procedures. Exclusion was applied when a diagnosis code associated with the HTO procedure was atypical and did not match with the 77.87 ICD-9-CM code.

### 2.2. Calculation Methodology

A series of descriptive statistical analyses was carried out by means of the R program (v4.4.1), a software environment for statistical computing and graphics.

### 2.3. Data Collection and Statistical Analysis

For categorical variables, frequency and percentage are provided; for continuous variables, means and standard deviations are provided. Incidence rates were calculated by dividing the number of annual cases by the size of the population, as reported each year by ISTAT, a legally required electronic national population registry.

## 3. Results

### 3.1. Demographics

From 2001 to 2016, 34,402 HTOs were performed in Italy, with a cumulative incidence of 3.6 cases per 100,000 residents. About 9.5% of patients required intervention for a trauma, and in 94.2% of cases, the intervention was the charge of the SSN.

**Figure 1:**
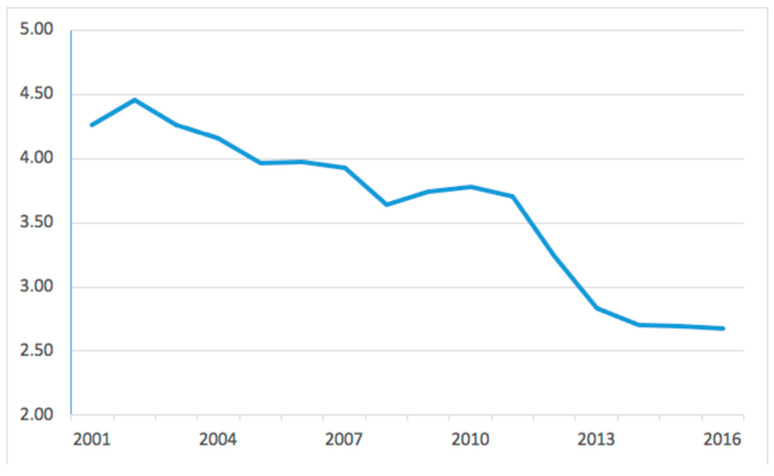
Incidence of high tibial osteotomies per 100,000 residents from 2001 to 2016 in Italy.

The incidence trend was decreasing, from a maximum of 4.5 in 2002 to a minimum of 2.7 cases per 100,000 residents in 2016 (Figure 1).

The male/female ratio (M/F) was 1.5 and the mean age of patients was 44.2 ± 19.2 years. Males were significantly younger than females (43.3 ± 20.7 vs. 45.6 ± 17.7, respectively, *p* < 0.0001).

**Figure 2:**
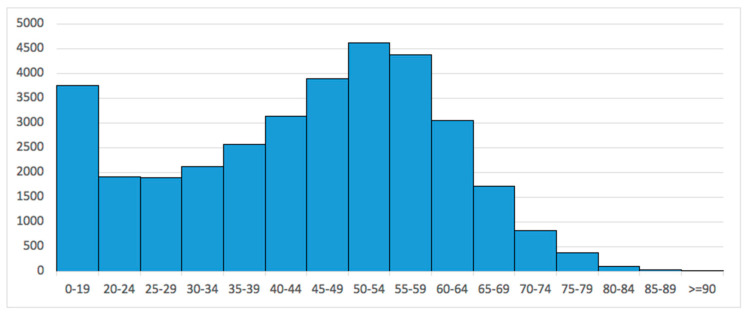
Number of high tibial osteotomies performed in Italy from 2001 to 2016, stratified by age groups.

Stratifying by age groups, the 50–54-year-old (13.4%) and the 55–59-year-old (12.7%) age groups required HTO the most. Pediatric patients largely required HTO, with 10.9% of patients in the 0–19-year-old age group (Figure 2).

### 3.2. Length of the Hospitalization

**Figure 3:**
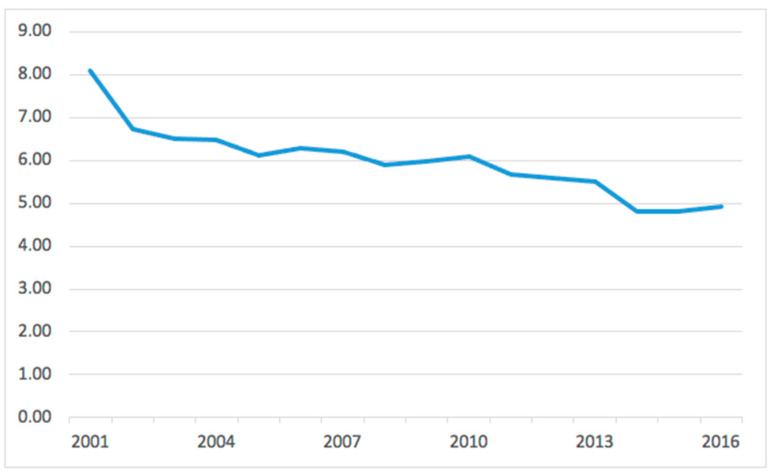
Average days of hospitalization per year.

The average length of hospitalization was 6.1 ± 7.3 days, with a decreasing trend (from 8.1 ± 7.6 days in 2001 to 4.9 ± 6.6 days in 2016). Median values halved from 2001 to 2016, decreasing from 6 to 3 days of hospital stay (Figure 3).

**Figure 4:**
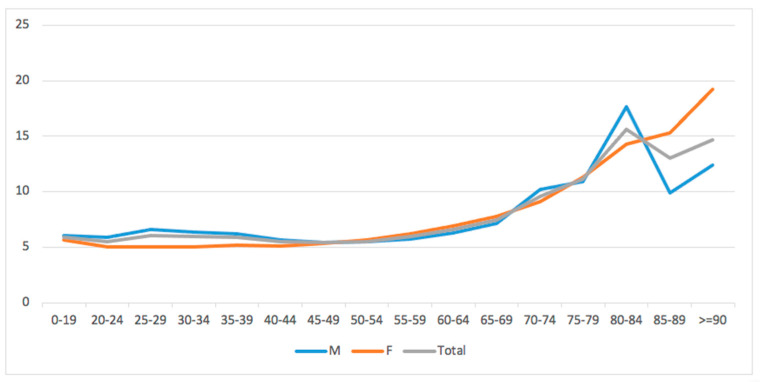
Average days of hospitalization stratified by age groups and gender.

No gender differences were found, while older patients (>70 years) showed on average the highest length of hospital stay (*p* < 0.001) (Figure 4).

### 3.3. Main Primary Diagnoses

The main primary diagnoses codes were “Varus knee” (736.42 ICD-9-CM code, 33.9%), “Osteoarthrosis, localized, primary, leg region” (715.16 ICD-9-CM code, 9.5%), “Osteoarthrosis, localized, secondary, leg region” (715.26 ICD-9-CM code, 6.1%), “Valgus knee” (736.41 ICD-9-CM code, 5.4%), “Fracture” (733.82 ICD-9-CM code, 5.8%), “Trauma of the lower limbs” (905.4 ICD-9-CM code, 5.4%), “Chondromalacia” (717.7 ICD-9-CM code, 3.6%).

## 4. Discussion

The current study is the first to present national trends for HTO in the Italian population. An official national register was used to assess the number of surgical procedures carried out in Italian public and private hospitals over the study period. The main finding of the present study was the number of HTO performed in Italy per 100,000 person-years. A dramatic decrease in the incidence of knee osteotomy has been shown over the years, almost halving from 2001 to 2016.

Regarding recent decades, many authors have reported similar decreasing trends of HTO in Western countries [28,29,30,31]. A 22-year Finnish population-based study reported a slight increase in incidence of knee osteotomies in patients <50 years, still confirming a general decline in the overall incidence of knee osteotomies [30]. Conversely, in Eastern countries, an opposite trend was observed. A Korean study reported an increase in HTO by 210% from 2009 to 2013, especially in patients aged from 55 to 64 years; a Japanese study reported an increase in HTO from 2.6% in 2007 to 5.5% in 2014. The improvement in TKA outcomes over recent decades and the evidence that UKAs have been used to treat individuals with less severe forms of arthritis may be the cause of the reduced need for osteotomies worldwide [32]. However, further studies are needed to investigate these continental variations in knee surgery preferences, taking into account the well-known specific features of knee anatomy in Asian people [33,34,35,36].

The present study showed that Italian males more frequently undergo HTO. Moreover, males are on average younger than females when undergoing surgery. Many orthopedic diseases have shown a gender prevalence [37,38,39,40,41,42]; varus deformity, with consequent medial compartment OA, is typically more common in men [43,44] and it may justify the M/F discrepancy in Italian patients requiring HTO. Furthermore, female gender was found to be an independent risk factor for failure in a 2019 study by Keenan et al. that examined 111 HTO patients with an average follow-up length of 12 years [45].

The age-specific subanalysis of patients included in the present study revealed that HTO is largely performed in pediatric patients (0–19 years of age class). The most common cause of genu varum requiring HTO in the pediatric setting is Blount disease; patients typically present in their second decade of life with severe genu varum, procurvatum, and internal tibial torsion [46,47]. Standard management for “adolescent tibia vara” (Blount disease) is proximal tibial osteotomy with or without associated fibular osteotomy [48,49,50,51]. However, recurrence of varus deformity after HTO in Blount disease has been described [52,53].

Data showed that from the age class of 20–24 years to the age class of 50–54 years, there were increasing requests for HTO in the Italian population. In those aged >60 years, the incidence of knee osteotomy progressively decreased. These results are in line with the current literature. An epidemiological analysis in Japan showed more requests for tibial osteotomy in patients <55 years, whereas UKA seemed to be preferred in patients >55 years [54]; in Korea, the largest increase in incidence of HTO and UKA was observed in the 55–64-year-old age group, with TKA becoming predominant in patients >75 years [55]. These trends, similar among countries, may have been influenced by reports suggesting that HTO is more appropriate for younger and active patients, whereas UKA is preferrable in elderly patients with lower activity levels [10,12,31]. It seems evident from the literature reported and from results of the present study that knee osteotomy plays a role in delaying knee OA progression, whereas knee arthroplasty represents the final option for osteoarthritic knees.

From 2001 to 2016, a trend toward shorter hospital stays for HTO procedures was seen. Hospitals frequently reduce duration of stay over time for economic reasons, which most likely explains the disparity. However, this claim is unsupported by any evidence.

The analysis of the primary diagnosis codes revealed a clear predominance of varus knee deformity and knee osteoarthritis as the leading causes requiring HTO in the Italian population. Accordingly, although valgus knee can also be managed with medial closing-wedge HTO, it is extremely more common to perform a lateral closing-wedge DFO. It is known that varus alignment may determine medial compartment knee osteoarthritis, and a correction osteotomy may interfere with this pathologic process [56,57]. Timing of surgery and treatment choice, particularly HTO vs. UKA, are still a matter of controversy in the orthopedic literature.

Noteworthy is that, when conducting epidemiologic studies on trends for consecutive years, it should always be taken into account that possible emergency situations may influence the incidence and hospitalization rates of orthopedic interventions. Moldovan et al. showed that the COVID-19 pandemic severely affected the volume of hip and knee arthroplasty in Romania, with unfavorable financial implications [58]. For this reason, it is important to continually collect clinical data and analyze how trends vary under the influence of external factors [58].

The study presents a list of limitations. First of all, the ICD-9-CM is the source for all reported diagnoses and procedures, based on administrative data from various hospitals and areas. Finding incorrect diagnoses or coding errors might be challenging given the large number of hospitals involved. In order to mitigate potential errors, we have stated our inclusion and exclusion criteria. The fact that hospitalizations in Italy’s healthcare system are anonymous and patients are not issued a unique ID number means that there are no outcome scores in this study. In other words, patients who had many procedures may have been counted more than once. Third, because ICD-9 classification was performed by surgeons, there can be variations between observers.

## 5. Conclusions

Overall, 34,402 HTOs were performed over the study period in Italy. The incidence of knee osteotomy over the years has almost halved from 2001 to 2016. Interestingly, HTO is largely performed in pediatric patients in order to correct alignment deformities. In line with the literature, results showed that from the 20–24-year-old age class to the 50–54-year-old age class, there were increasing requests for HTO, whereas in those aged >60 years, the incidence of knee osteotomy progressively decreased. As expected, varus deformity and knee osteoarthtitis were the leading diagnoses managed with HTO. The evident decline in HTO performed over the years in Italy seems to reflect a minor role for knee osteotomy in the management of knee OA, as it seems to be primarily reserved for younger male patients. Further studies are necessary in order to define the best standards of care for knee OA and the future of knee osteotomies. Moreover, it is important to continually collect clinical data and analyze how trends vary under the influence of external factors.

## Data Availability

The datasets used and/or analyzed during the current study are available from the corresponding author on reasonable request. The access to the database is on request. All data were obtained by the Direzione Generale della Programmazione Sanitaria—Banca Dati SDO of the Italian Ministry of Health.
